# Expression of STOML2 promotes proliferation and glycolysis of multiple myeloma cells via upregulating PAI-1

**DOI:** 10.1186/s13018-021-02819-2

**Published:** 2021-11-13

**Authors:** Hongxia Zhang, Guangsheng Wu, Junjian Feng, Xiaohong Lu, Ping Liu

**Affiliations:** 1 Department of Hematology, First Affiliated Hospital, School of Medicine, Shihezi University, Shihezi City, 832008 Xinjiang Uygur Autonomous Region China; 2 Department of Intensive Care Unit, Luzhou People’s Hospital, Luzhou City, 646000 Sichuan Province China; 3 Department of Hematology, Luzhou People’s Hospital, Luzhou City, 646000 Sichuan Province China; 4 Department of Pathology, Luzhou People’s Hospital, Section 2 of JiuGu Avenue, Jiangyang District, Luzhou City, 646000 Sichuan Province China

**Keywords:** STOML2, Cell proliferation, Glycolysis, Multiple myeloma, PAI-1

## Abstract

**Background:**

This study aimed to investigate the effects of STOML2 and the relationship between STOML2 and PAI-1 in the development of multiple myeloma (MM).

**Methods:**

Cell proliferation was tested using CCK-8 assay and cell colony formation assay. Glucose consumption, lactate production and ATP level were measured using commercial kits. The mRNA and protein expression were assessed using quantitative real-time polymerase chain reaction (qRT-PCR) and western blotting, respectively.

**Results:**

Both mRNA and protein expression of STOML2 were upregulated in MM patients compared to healthy volunteers. CCK-8 and colony formation assays demonstrated that STOML2 silencing inhibited cell proliferation in MM cells. Knockdown of STOML2 reduced glucose consumption, lactate production and ATP/ADP ratios. STOML2 silencing by shSTOML2 led to reduced PAI-1 expression. Overexpression of PAI-1 reversed the inhibitory effects of shSTOML2 on MM cell growth.

**Conclusion:**

Results from this study demonstrated that STOML2 silencing inhibits cell proliferation and glycolysis through downregulation of PAI-1 expression, suggesting a new therapeutic target for MM.

## Background

Multiple myeloma (MM) is one of the most common hematologic malignancies, accounting for 10–15% hematological neoplasms and ~ 1% of all cancers [1]. MM developed from monoclonal gammopathy of uncertain significance and mainly diagnosed in elderly [1]. Several risk factors have been postulated to contribute to MM occurrence, such as chronic infection, obesity, and exposure to pesticides and benzol [1]. The clinical symptoms of MM include anemia, bone pain, fatigue and weight loss [2]. Current first-line treatment for MM is stem-cell transplantation [3]. For patients who are not eligible for stem-cell transplantation or recurrent MM, their treatment options include chemotherapy and are usually prescribed with new targeted drugs, such as proteasome inhibitors, and immunomodulatory agents [3].

The etiology of MM remains unclear. The genetic alterations and bone marrow microenvironment have been identified to contribute to the progression of MM [2], providing novel biomarkers and therapeutic targets for novel drug discovery. Plasminogen activator inhibitor-1 (PAI-1) is a member of serpin super-family and is upregulated in pathological processes, including thrombosis, vascular disease and various cancers [4]. The expression of PAI-1 was found to be elevated in MM patients and is associated with clinical outcomes in MM patients [5]. PAI-1 has been reported to regulate cancer cell adhesion and invasion, and can induce tumor vascularization [4]. Thus, this prompts the speculation that manipulation of PAI-1 expression may be a potential treatment strategy for different cancers, including MM.

Stomatin-like protein-2 (STOML2) is a family member of stomatin-like proteins, which cause the overhydrated hereditary stomatocytosis [6]. Previous study has shown that overexpression and colocalization of STOML1 and STOML2 at the cell membrane and in the cytoplasm is positively related to the T1 and T2 stages of oral squamous cell carcinoma [7]. Moreover, upregulation of STOML2 has been identified in many cancers, including gastric adenocarcinoma, glioma and cervical cancer [8–10], indicating a role of STOML2 in cancer progression. However, the effects of STOML2 in MM remain unknown. Thus, this study aimed to investigate the effects of STOML2 and the relationship between STOML2 and PAI-1 in the development of multiple myeloma (MM), providing a new prognostic marker and therapeutic target for MM.

## Methods

### Human serum collection

Human serum was collected from multiple myeloma patients (*n* = 40) and healthy volunteers (*n* = 25) at First Affiliated Hospital, School of Medicine, Shihezi University. Written informed consents were collected from all patients for sample analysis and data publication. This study complied with the Declaration of Helsinki [11] and the protocols were approved by the Ethics Committee of First Affiliated Hospital, School of Medicine, Shihezi University.

### Cell culture and transfection

Human normal plasma cells (nPCs) and human multiple myeloma cell lines (RPMI-8226, NCI-H929 and U266 cells) were obtained from Wuhan Procell Life Science &Technology Co., Ltd. (Procell, China). RPMI-1640 medium (Procell) was supplemented with 10% fetal bovine serum (FBS, Merck KGaA, Germany) and 1% penicillin/streptomycin/amphotericin B Solution (100×, Merck KGaA). All cells were cultured in complete RPMI-1640 medium (normal culture medium) at 37 °C with 5% CO_2_.

Short hairpin RNAs of STOML2 (shSTOML2-1# and hSTOML2-2#) and scrambled RNAs (shNC) were purchased from Shanghai GenePharma Technology Co., Ltd. (GenePharma, China). Full coding sequence of PAI-1 or control sequence (NC, GenePharma) was cloned into pcDNA 3.1 plasmids to generate pcDNA 3.1-PAI-1 and pcDNA 3.1-NC vector, respectively. Cell transfection was carried out using RNAifenctin transfection reagent or DNAfectin 2400 (AmyJet, China) according to the manufacturer’s instructions.

### CCK-8 assay and cell colony formation assay

Cell proliferation was examined using CCK-8 assay and cell colony formation assay. For CCK-8 assay, 1 × 10^3^ transfected cells/well were seeded onto 96-well plates and cultured for 48 h at 37 °C. CCK-8 assay was conducted using the commercial CCK-8 kit (Abcam, UK) according to the manufacturer’s instructions. For colony formation assay, the transfected cells were seeded in 6-well plates at a density of 50 cells/well. After 2 weeks of incubation at 37 °C with 5% CO_2_, cells were fixed with 4% paraformaldehyde (Aladdin, China) for 15 min and then stained with 0.1% crystal violet (Solarbio, China) for 20 min. The stained cell colonies were imaged and counted under an inverted microscope (Leica, Germany).

### Measurement of glucose consumption, lactate production and ATP levels

Cells were seeded onto 6-well plates at a density of 1 × 10^6^ cells/well. At 48 h post-transfection, glucose consumption, lactate production and ATP level were measured using Glucose Uptake Colorimetric Assay Kit (Merck KGaA), L-Lactate Assay Kit (Abcam) and ADP/ATP Ratio Assay Kit (Abcam), respectively, according to manufacturers’ protocols.

### Quantitative real-time polymerase chain reaction (qRT-PCR)

Total RNA was isolated and purified using Purelink RNA mini kits (Thermo Fisher, USA). A total of 200 ng of RNA was reversely transcribed to cDNA using SuperScript IV Reverse Transcriptase (Thermo Fisher). cDNA was amplified using SYBR® Green Quantitative RT-qPCR Kit (Sigma-Aldrich, USA) and relative RNA expression was quantified using 2^−ΔΔCT^ method. Primer sequences used in this study are as follows: β-actin forward, 5′-GCTGCATTTAGTGGCCTCATT-3′ and reverse, 5′-GCAAGGCATAACCTGATGTGG-3′; STOML2 forward, 5′-GGCTGTGACTCTCGACAATGT-3′ and reverse, 5′-CACACCGTAGCTTGCCTTGTA-3′; and PAI-1 forward, 5′-GTCCACTTCCACGTCATGC-3′ and reverse, 5′-GGGAGGTAGACACGGGGAT-3′.

### Western blotting

Proteins were lysed and extracted using ProteoPrep® Total Extraction Sample Kit (Merck KGaA) and the concentration of protein was measured using BCA Protein Assay Kit (ABCam). A total of 5 μg of protein per well was loaded into SDS-PAGE gel (BioRad, USA) and separated by electrophoresis. The separated protein was then transferred onto PVDF membranes (Merck KGaA, Germany). After blocking the membranes with 5% of fat-free milk for 1 h at room temperature, the membranes were probed using appropriate primary antibodies and incubated overnight at 4 °C, followed by incubation with secondary antibodies the next day for 2 h at room temperature. Then, protein signals were detected using Pierce™ ECL Western Blotting Substrate (Thermo Fisher, USA). Primary antibodies (Thermo Fisher) used in this study are as follows: STOML2 (MA5-26962, 1:1000 dilution), Bcl2 (MA1-26233, 1:500 dilution), Bax (PA5-11378, 1:1000 dilution), Clv-PARP (MA5-37258, 1:1000 dilution), PCNA (13-3940, 1:2000 dilution), PAI-1 (PA5-35128), GLUT1 (PA1-46152, 1:500 dilution), HK2 (PA5-17257, 1:15000 dilution), LDHA (MA5-17247, 1:2000 dilution) and β-actin (MA1-140, 1:3000 dilution).

### Statistical analysis

Statistical analysis was carried out using GraphPad Prism 7.0 (GraphPad, USA). All results are presented as mean ± SD. Statistical difference was performed using unpaired student *t* test (two groups) and one-way univariate analysis of variance with post hoc Tukey’s tests (multiple groups). A *p* value of less than 0.05 indicated statistical significance.

## Results

### STOML2 is overexpressed in MM sera and cell lines.

**Fig. 1:**
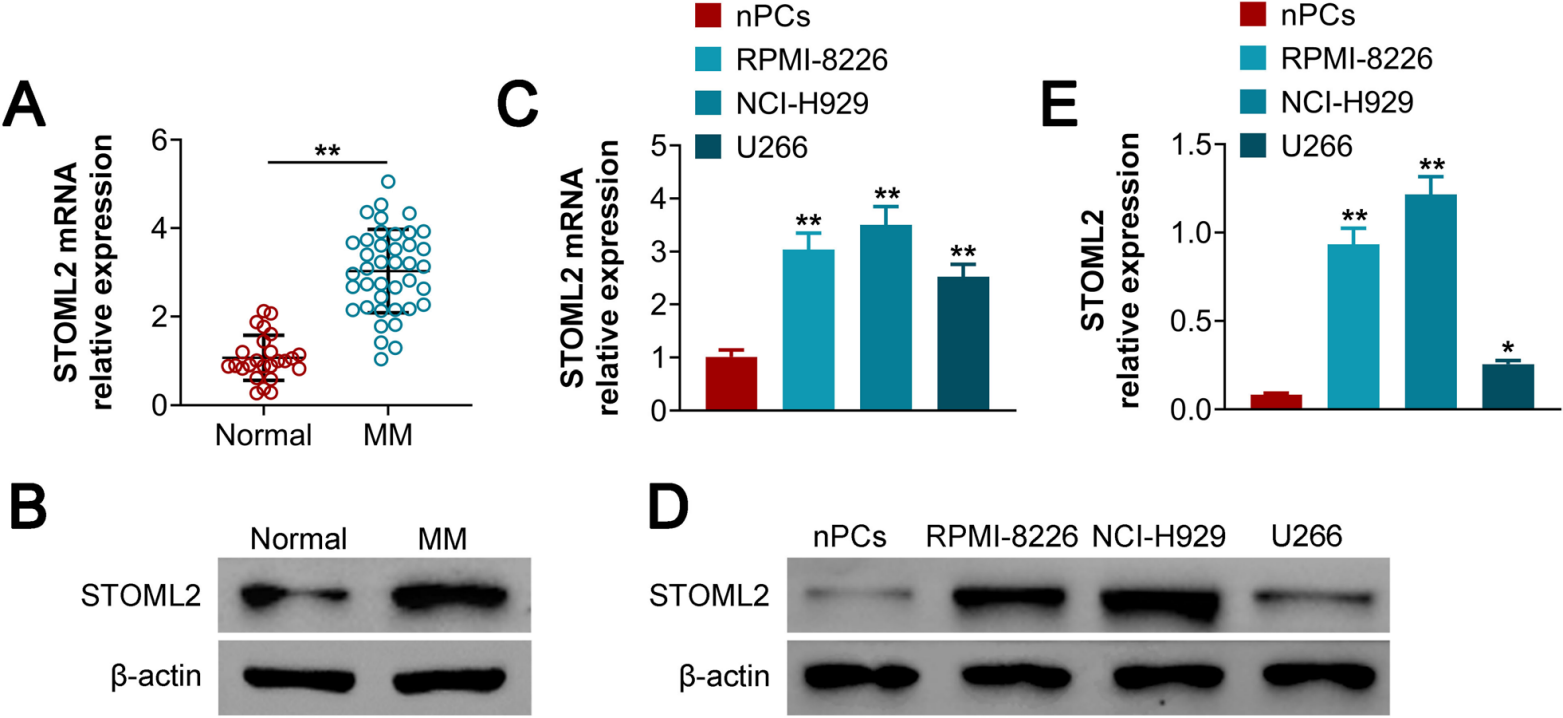
STOML2 is overexpressed in MM sera and cell lines. **A** The expression of STOML2 mRNA is upregulated in MM patients; **B** the expression of STOML2 protein is upregulated in MM patients; **C** the expression of STOML2 mRNA is upregulated in MM cell lines; **D** the expression of STOML2 protein is upregulated in MM cell lines; **E** statistical analysis shows that the expression of STOML2 protein is upregulated in MM cell lines. *p < 0.05 versus (vs) nPCs; **p < 0.01 vs Normal or nPCs; ***p < 0.005 vs Normal or nPCs. MM: multiple myeloma

The transcript (Fig. 1A) and protein levels (Fig. 1B) of STOML2 were found to be upregulated in the sera of MM patients compared with that of healthy controls (Fig. 1A). In addition, STOML2 was also increased in human MM cell lines, at both the mRNA (Fig. 1C) and protein levels (Fig. 1D, E). These results demonstrated that STOML2 is upregulated in MM.

### Knockdown of STOML2 inhibits cell growth in MM.

**Fig. 2:**
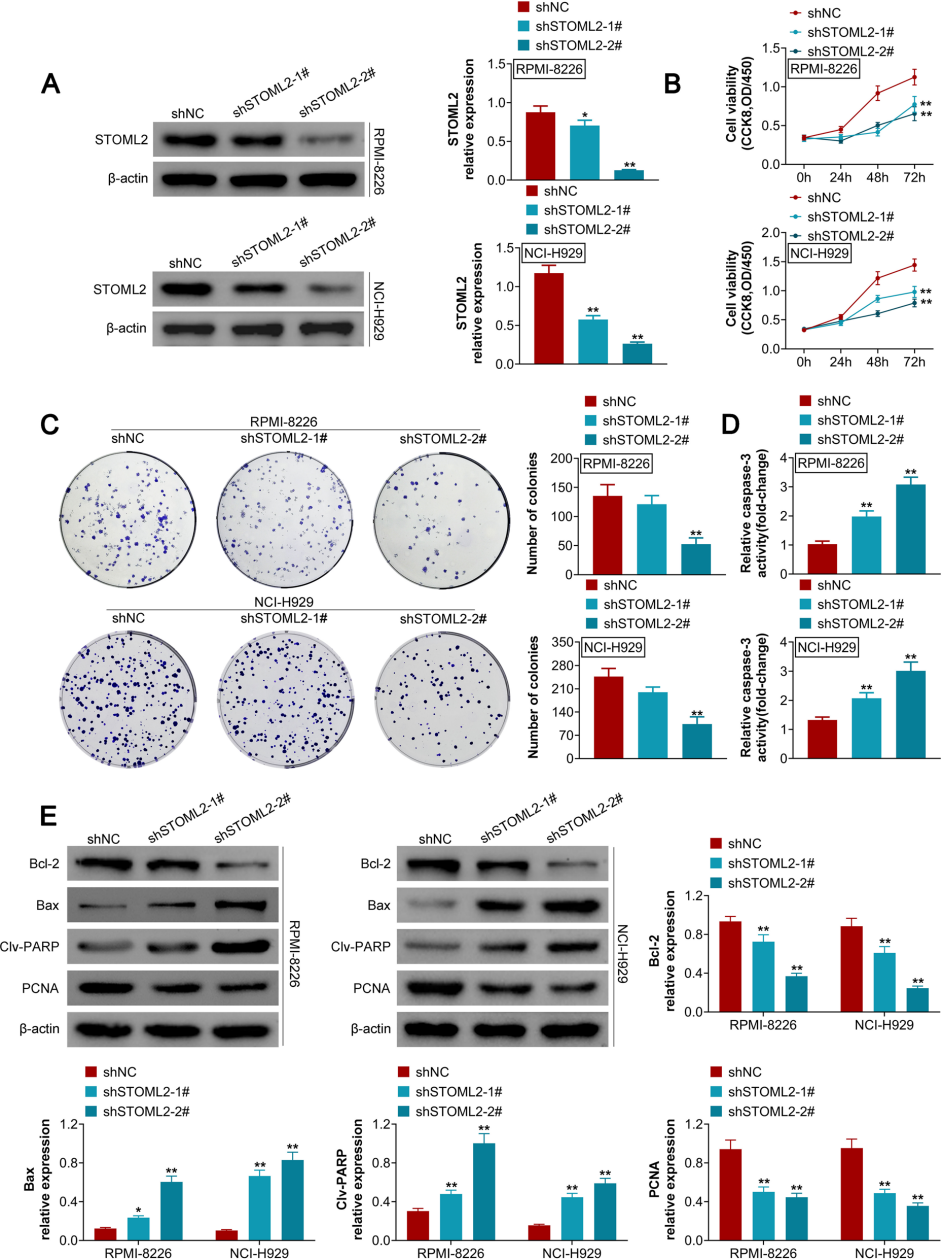
Knockdown of STOML2 inhibits MM cell growth. **A** Protein expression of STOML2 is reduced by shSTOML2-1# and shSTOML2-2# in MM cell lines; **B** MM cell viability is reduced by shSTOML2-1# and shSTOML2-2#; **C** the number of cell colonies is reduced by shSTOML2-1# and shSTOML2-2# in MM cell lines; **D** the activity of Caspase-3 is increased by shSTOML2-1# and shSTOML2-2# in MM cell lines; **E** the protein expression of Bcl2 and PCNA is downregulated, and the protein expression of Bax and Clv-PARP is upregulated by shSTOML2-1# and shSTOML2-2# in MM cell lines. *p < 0.05 vs shNC; **p < 0.01 vs shNC. shSTOML2: short hairpin RNAs of STOML2; shNC: scrambled RNA of shSTOML2

STOML2 expression was significantly reduced by shSTOML2 in RPMI-8226 and NCI-H929 cells (Fig. 2A), with shSTOML2-2# resulted in a better knockdown efficiency compared to shSTOML2-1# (Fig. 2A). Results from the CCK-8 assays demonstrated that STOML2 silencing led to decreased cell viability in human MM cell lines (Fig. 2B). The number of cell colonies was significantly reduced by shSTOML2-2# but not shSTOML2-1# (Fig. 2C). In addition, increased Caspase-3 activity was observed in MM cells transfected with shSTOML2-1# and shSTOML2-2# (Fig. 2D). STOML2 knockdown resulted in reduced protein expression of Bcl-2 and PCNA, and increased Bax and Clv-PARP levels (Fig. 2E). These results indicated that STOML2 silencing inhibits cell proliferation and promotes cell apoptosis in MM.

### STOML2 silencing represses abnormal glycolysis and downregulates PAI-1 expression in MM.

**Fig. 3:**
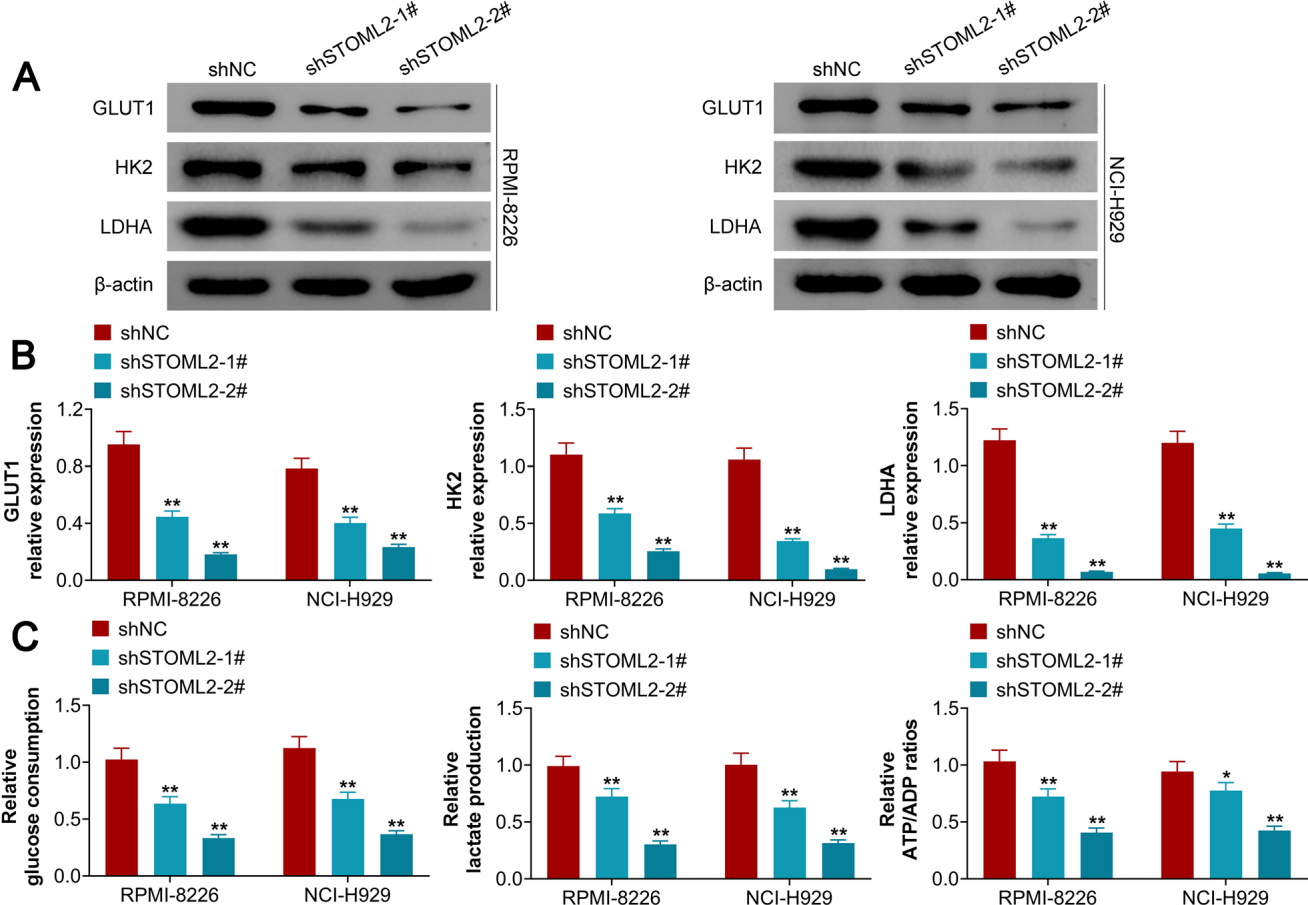
Knockdown of STOML2 represses abnormal glycolysis in MM. **A** The protein expression of GLUT1, HK2 and LDHA is downregulated by shSTOML2-1# and shSTOML2-2# in MM cell lines; **B** statistical analysis shows that the protein expression of GLUT1, HK2 and LDHA is downregulated by shSTOML2-1# and shSTOML2-2# in MM cell lines; **C** the relative glucose consumption, lactate production and ATP/ADP ratio are reduced by shSTOML2-1# and shSTOML2-2# in MM cell lines. *p < 0.05 vs shNC; **p < 0.01 vs shNC

**Fig. 4:**
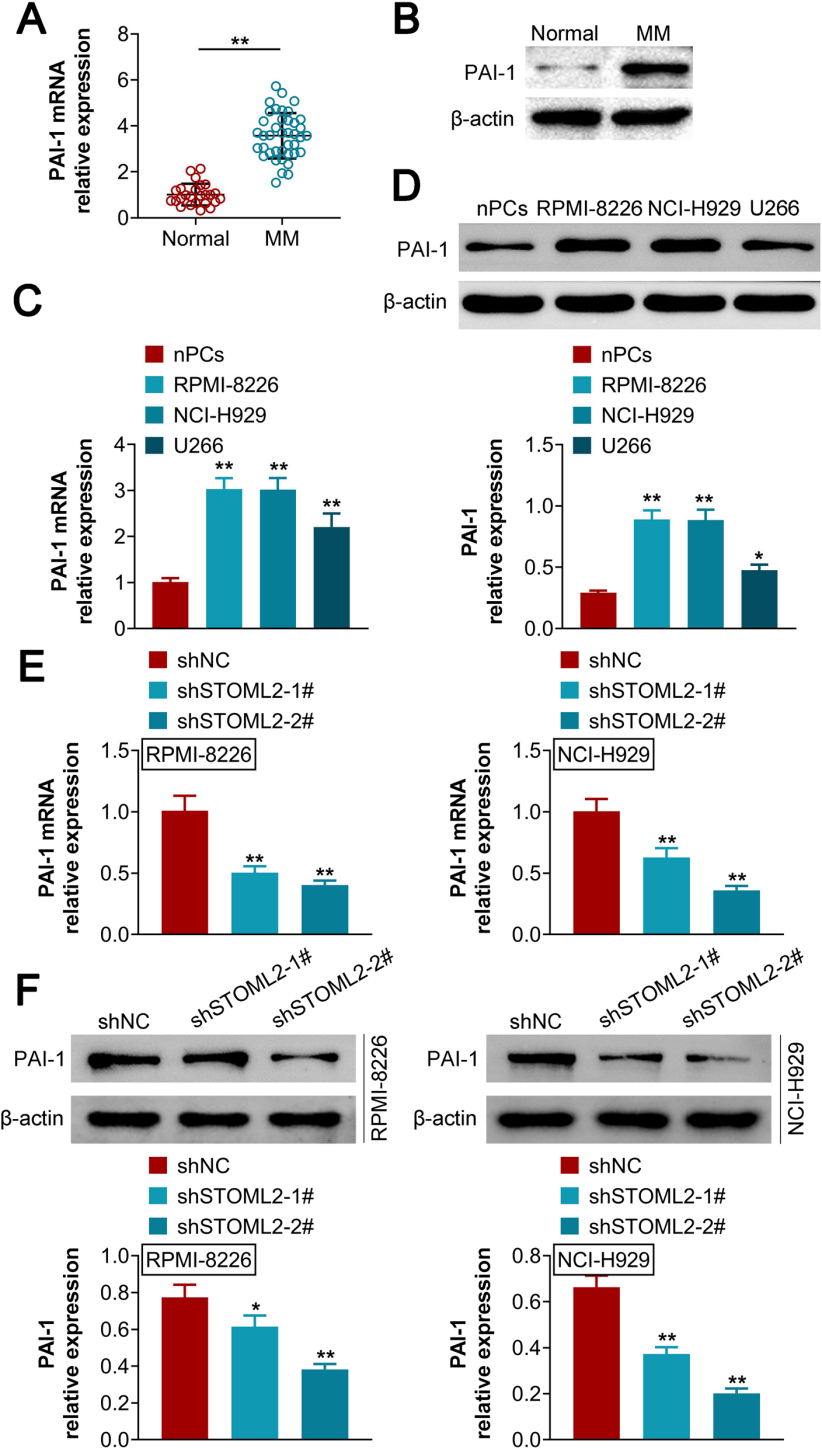
Expression of PAI-1 is regulated by STOML2 in MM. **A** The mRNA expression of PAI-1 is downregulated y shSTOML2-1# and shSTOML2-2# in MM cell lines; **B** the protein expression of PAI-1 is downregulated y shSTOML2-1# and shSTOML2-2# in MM cell lines. *p < 0.05 vs shNC; **p < 0.01 vs shNC

STOML2 silencing inhibited the protein expression of glucose transporter type 1 (GLUT1) and hexokinase 2 (HK2) but elevated the levels of lactate dehydrogenase A (LDHA) (Fig. 3A, B). Furthermore, our results demonstrated that STOML2 knockdown led to a significant reduction in glucose consumption, lactate production and ATP levels (Fig. 3C). Both mRNA and protein levels of PAI-1 were found to be upregulated in MM serum (Fig. 4A, B) and MM cell lines (Fig. 4C, D), compared to their control counterparts. Interestingly, STOML2 silencing by shSTOML2 reduced PAI-1 expression, at both the mRNA (Fig. 4E) and protein levels (Fig. 4F) in MM cell lines. These data indicated that knockdown of STOML2 represses abnormal glycolysis and downregulates PAI-1 expression in MM.

### PAI-1 reverses STOML2 silencing-mediated tumor cell growth inhibition in MM.

**Fig. 5:**
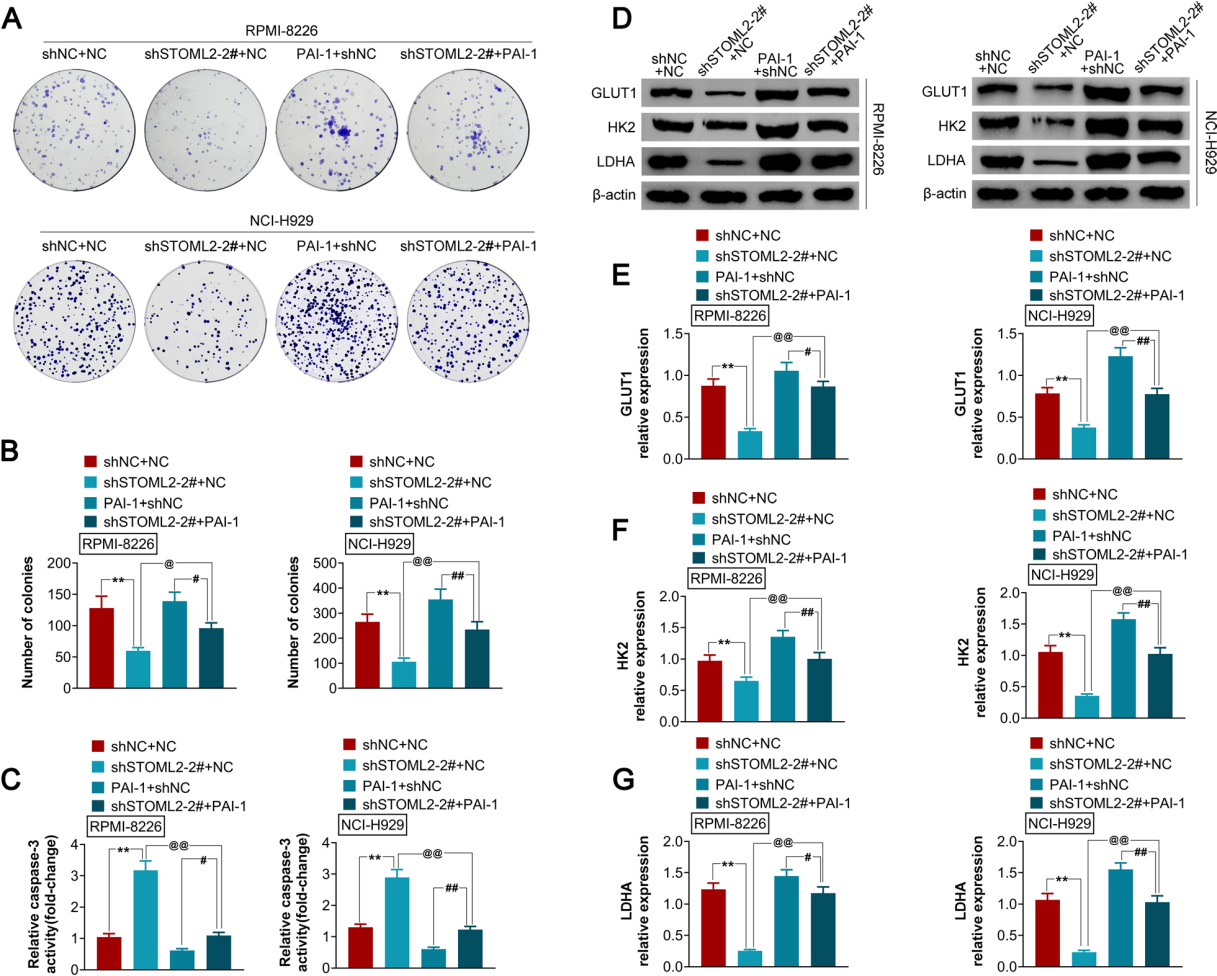
PAI-1 reverses STOML2 silencing-mediated tumor cell growth inhibition in MM. **A** PAI-1 reverses shSTOML2-mediated cell proliferation inhibition; **B** statistical analysis shows that PAI-1 reverses shSTOML2-mediated cell proliferation inhibition; **C** PAI-1 reverses shSTOML2-mediated increase in Caspase-3 activity; **D** PAI-1 reverses shSTOML2-mediated decrease in GLUT1, HK2 and LDHA; **E**–**G** Statistical analyses show that PAI-1 reverses shSTOML2-mediated downregulation of GLUT1 **E**, HK2 **F**, and LDHA **G**. **p < 0.01 vs shNC; ^@^*p* < 0.05 vs shSTOML2-2# + NC; ^@@^*p* < 0.01 vs shSTOML2-2# + NC; ^#^*p* < 0.05 vs PAI-1 + shNC; ^##^*p* < 0.05 vs PAI-1 + shNC. PAI-1: pcDNA3.1 plasmid containing full coding sequence of PAI-1; NC: scrambled sequence of PAI-1

Next, we demonstrated that inhibited cell proliferation caused by shSTOML2 could be reversed by PAI-1 overexpression in MM cell lines (Fig. 5A, B). The caspase activity was increased by shSTOML2 and decreased by PAI-1 overexpression. The changes in caspases were attenuated by co-transfection of shSTOML2 and PAI-1 plasmid (Fig. 5C). In cells co-transfected with shSTOML2 and PAI-1 plasmid, the protein expression of GLUT1, HK2 and LDHA was higher than that of cells transfected with shSTOML2 alone but lower than that of PAI-1 plasmid transfected cells (Fig. 5D–G), suggesting that PAI-1 reverses STOML2 silencing-mediated tumor cell growth inhibition in MM.

## Discussion

Owing to a better understanding of genetic alterations underlying MM and advancements in targeted therapy, the clinical outcomes have been improved in MM patients in the past decades [12]. However, considering the heterogeneity in clinical features of MM, mechanisms underlying the pathogenesis of MM and newly discovered causative gene mutations should be further interrogated to improve the prognosis of MM [12]. In this study, STOML2 was found to be upregulated in MM patients. Knockdown of STOML2 inhibited cell growth and glycolysis in MM cell lines. STOML2 silencing downregulated the expression of PAI-1 and PAI-1 overexpression attenuated the effects of shSTOML2 in MM, suggesting that the effects of STMOL2 were mediated through PAI-1. These findings demonstrated that STOML2 and PAI-1 promote the development and progression of MM, providing novel targets for future drug discovery and treatment strategy for MM.

To date, only a number of reports indicate that STOML2 contributes to tumorigenesis in cancer progression and metastasis. For example, through regulating PINK1-mediated mitophagy, STOML2 was found to promote cancer metastasis and lenvatinib resistance in hepatocellular carcinoma [13]. STOML2 has been shown to increase cell migration and invasion through activation of nuclear factor kappa B signaling pathway in human live cancer [14]. This study has demonstrated for the first time that STOML2 was upregulated in MM. STOML2 silencing inhibited cell proliferation and accelerated cell apoptosis. These findings indicate that expression of STOML2 is associated with tumorigenesis in MM, thereby suggesting that STOML2 may be a potential prognostic maker for MM.

Glucose is the essential energy source for maintenance of the normal function in human organs and tissues [15]. It is found that cancer cells prefer aerobic glycolysis even under the condition where oxygen supply is sufficient [15]. During aerobic glycolysis, glucose consumption is increased and converted to lactic acid, resulting in an elevation of lactate production [15]. In the present study, inhibition of STOML2 reduced glucose consumption and lactate production, indicating that STOML2 is involved in aerobic glycolysis in MM. Aerobic glycolysis also promotes adenosine-diphosphate (ADP) conversion to adenosine-triphosphate (ATP) in the cytoplasm [16]. In this study, ATP/ADP ratio was reduced and the expression of GLUT1, HK2 and LDHA was downregulated following STOML2 knockdown, further confirming that STOML2 is involved in aerobic glycolysis in MM.

As mentioned in the introduction, PAI-1 regulates cancer cell adhesion and invasion, and induces tumor vascularization [4]. In the present study, suppression of STOML2 reduced the expression of PAI-1 mRNA and protein, implying that STOML2 may be involved in tumor angiogenesis. There is an increased risk of thromboembolic events in MM due to impaired fibrinolysis [17]. Increased PAI-1 also contributes to impaired fibrinolysis [17]. Therefore, STOML2 might also increase the incidence of thromboembolic events in MM. In addition, our findings have demonstrated that the effects caused by STOML2 silencing could be reversed by overexpressing PAI-1. These results have shown that STOML2 promotes cell proliferation and aerobic glycolysis through regulation of PAI-1 expression.

## Conclusion

In conclusion, upregulation of STOML2 was observed in MM sera and MM cell lines. STOML2 silencing by short hairpin RNA inhibits cell proliferation and aerobic glycolysis in MM cell lines and this process is reversed through PAI-1 overexpression. These results demonstrate that STOML2 promotes cell proliferation and aerobic glycolysis in MM through regulation of PAI-1 expression, providing a new mechanism of MM progression and a novel therapeutic target for drug discovery.

## Data Availability

All data generated or analyzed during this study are included in this published article.

## Abbreviations

MM: Multiple myeloma
qRT-PCR: Polymerase chain reaction
shSTOML2: Short hairpin RNA of STOML2
PAI-1: Plasminogen activator inhibitor-1
